# Pupillary Responses for Cognitive Load Measurement to Classify Difficulty Levels in an Educational Video Game: Empirical Study

**DOI:** 10.2196/21620

**Published:** 2021-01-11

**Authors:** Hugo Mitre-Hernandez, Roberto Covarrubias Carrillo, Carlos Lara-Alvarez

**Affiliations:** 1 Center for Research in Mathematics Zacatecas Mexico; 2 Center for Research and Advanced Studies of the National Polytechnic Institute, Tamaulipas Ciudad Victoria Mexico

**Keywords:** video games, pupil, metacognitive monitoring, educational technology, machine learning

## Abstract

**Background:**

A learning task recurrently perceived as easy (or hard) may cause poor learning results. Gamer data such as errors, attempts, or time to finish a challenge are widely used to estimate the perceived difficulty level. In other contexts, pupillometry is widely used to measure cognitive load (mental effort); hence, this may describe the perceived task difficulty.

**Objective:**

This study aims to assess the use of task-evoked pupillary responses to measure the cognitive load measure for describing the difficulty levels in a video game. In addition, it proposes an image filter to better estimate baseline pupil size and to reduce the screen luminescence effect.

**Methods:**

We conducted an experiment that compares the baseline estimated from our filter against that estimated from common approaches. Then, a classifier with different pupil features was used to classify the difficulty of a data set containing information from students playing a video game for practicing math fractions.

**Results:**

We observed that the proposed filter better estimates a baseline. Mauchly’s test of sphericity indicated that the assumption of sphericity had been violated (χ^2^_14_=0.05; *P*=.001); therefore, a Greenhouse-Geisser correction was used (ε=0.47). There was a significant difference in mean pupil diameter change (MPDC) estimated from different baseline images with the scramble filter (*F*_5,78_=30.965; *P*<.001). Moreover, according to the Wilcoxon signed rank test, pupillary response features that better describe the difficulty level were MPDC (*z*=−2.15; *P*=.03) and peak dilation (*z*=−3.58; *P*<.001). A random forest classifier for easy and hard levels of difficulty showed an accuracy of 75% when the gamer data were used, but the accuracy increased to 87.5% when pupillary measurements were included.

**Conclusions:**

The screen luminescence effect on pupil size is reduced with a scrambled filter on the background video game image. Finally, pupillary response data can improve classifier accuracy for the perceived difficulty of levels in educational video games.

## Introduction

### Overview

An *educational video game* (EVG) is a video game that provides learning or training value to the player. Potential contributions of video games cover each of the three main fields of psychology: the affective (awakening feelings), the connate (aggressive or impulsive behavior), and the cognitive (learning-related skills) [1].

Video games have been demonstrated to be effective for improving working memory, mental rotation skills, and geometry performance [2]. Some of the effective features of educational video games include a clear goal, an adequate level of difficulty, quick-moving stimuli, and integrated instructions [3].

Several works have used EVGs to foster fraction understanding and to assess students [4,5]. However, our research focuses on the cognitive load (mental effort) generated by reasoning tasks [6] about math fractions; this is a direct way to measure the difficulty perceived by the EVG's player.

Video game difficulty refers to the amount of skill required by the player to progress through the game experience. Studying how to set an adequate difficulty level has attracted particular attention in the educational video games field [7,8]. Basic approaches to setting difficulty include allowing users to manually select levels and increasing the difficulty at a steady rate over the course of the game, with earlier levels being easier and later levels being harder [9]. Manually adapting difficulty or designing an incremental-difficulty solution could cause serious problems; for instance, the player may not know how they will perform before playing a given level, or the predefined change rate could be slower or faster than required by the player.

On the other hand, *dynamic difficulty adjustment* or *dynamic difficulty balancing* changes the game behavior according to the skill level of the players. For this purpose, the dynamic difficulty adjustment requires evaluation of the player's performance (through game scores, time, number of errors, player's decisions, etc) and adjustment of a set of game variables that regulate difficulty [10]. It has been shown that a dynamic approach that uses gamer behavior data presents better learning outcomes than an incremental difficulty approach [7].

As a step toward finding an imperceptible difficulty control, this paper proposes to use pupil dilation to detect very easy (or hard) activities. It is known that pupil dilation reflects activity in the brain as cognitive load—that is, the total amount of mental effort (information processing) induced by reasoning tasks or involving memory resources [6,11].

### Background

#### The Impact of Difficulty on Learning

**Figure 1:**
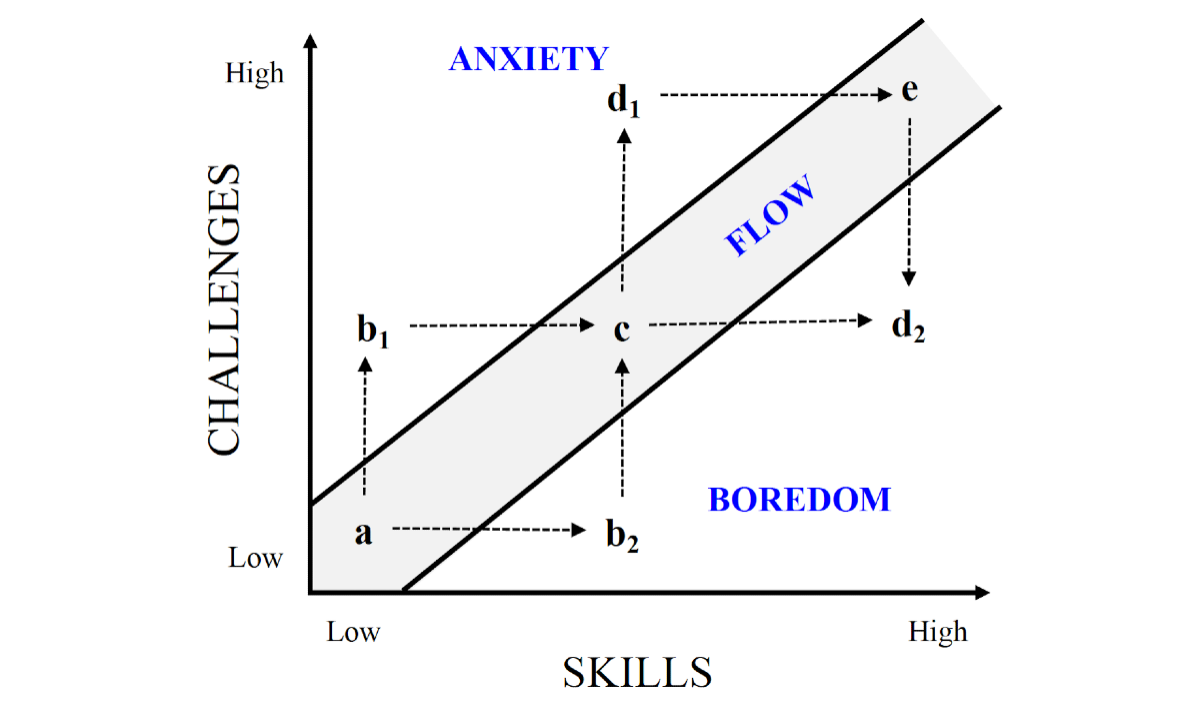
Flow experience model of Mihaly Csikszentmihalyi [12].

The flow experience model, proposed by Csikszentmihalyi [12], marks an achieved balance of arousal-increasing and arousal-decreasing processes. As shown in Figure 1, the flow model describes this balance in terms of the fit between perceived challenges and skills: an activity wherein challenges predominate increases arousal; an activity wherein skills predominate reduces arousal. Thus, a synchrony of challenges and skills permits a state of deep involvement, while the pitfalls of either over- or under-arousal (ie, anxiety or boredom) are avoided [12].

The dynamic flow passing through states *a* → *c* → *e* shown in Figure 1 is the optimal path for increasing difficulty. However, *b_1_* → *d_1_* are states of anxiety that demand new learning skills to return to optimal flow. Moreover, *b_2_* → *d_2_* are states of boredom that need more challenges to return to optimal flow. Several studies have supported that the rate of change of pupil diameter is related to task difficulty.

#### Pupillary Responses

The eye can be seen as a camera, with the pupil as the eye aperture, and it involves the iris activity [13]. The iris movement is controlled by the activity of two muscles, the dilator and the sphincter. Sphincter activation causes the pupil to constrict (ie, miosis), and this is largely under parasympathetic control, while the dilator muscle receives mostly sympathetic innervation and causes the pupil to dilate (ie, mydriasis) [14].

Light has a relevant role in the retina and the pupil response. The size of the iris determines the amount of light that is captured by the system. The ambient light level largely determines the steady-state size of the pupil. Rapid increments in light flux on the retina cause a brisk constriction of the pupil. This constriction will depend on the size of the light stimulus, its luminance contrast, onset temporal characteristics, and location in the visual field [14].

Health factors also affect pupillary responses. Pupillary constriction is decreased in major depression [15]. Schizophrenia is associated with a significant decline in working memory capacity, and an additional moderate decline is associated with aging, but pupillary responses evoked by a working memory task were not related to schizophrenia severity [16]. Among other factors, the consumption of caffeine or alcoholic beverages was associated with significant increases in pupil size [17,18]. Finally, pupil dilation can be caused by amphetamines and diphenhydramine, and pupil constriction by clonidine and opioids [19].

For good observation of pupil response during EVG tasks, all these conditions must be carefully observed in the experiment design.

#### Cognitive Load and Pupillary Response

The cognitive load (mental activity) imposed by tasks has a pupillary response, known as a task-evoked pupillary response (TEPR) [20]. TEPRs occurs shortly after the onset of a task and subside quickly after the mental activity is terminated. The TEPR depends on several factors; for instance, the response is greater for novice participants doing an arithmetic task than for an expert because novices require more mental effort [21]. Then, through pupillometry (measuring the pupil diameter), one can decide whether a challenge is adequate for the skills of a learner (Figure 1); that is, we can balance a video game to maximize the learning outcomes.

Pupil diameter is widely used to study cognitive load. Researchers have studied this relationship in different tasks, such as driving a vehicle while listening to a dialog, reasoning through math exercises, memorizing numbers, and perceiving visual stimuli [6,22,23].

Concerning industrial areas, cognitive load has been used in automotive and healthcare applications to optimize user's decision-making tasks [21,24]. Most studies in these fields are oriented to discover how to preserve attention and mental work on primary tasks and how to reduce it on secondary tasks to avoid critical errors. In addition, cognitive load has been used in video game studies without significant results, mainly due to changes in screen luminescence.

Playing EVG involves memorization and reasoning tasks that are associated with cognitive load. This paper uses pupillary response data to assess cognitive load in educational video games.

Beatty [6] points out that pupillary responses occur at short latencies following the onset of mental processing and subside quickly once processing is terminated. Most of the latency is due to slow iris muscle constriction. Different features have been used to evaluate cognitive load with pupillary responses such as mean pupil diameter change (MPDC), average percentage change in pupil size (APCPS), peak dilation (PD), and latency to peak (LP) [13,24-26].

#### Estimating Pupillary Responses

Individual differences in pupil size have been well documented; for example, pupil size decreases linearly as a function of age at all illuminance levels, and students high in cognitive ability have a larger pupil size [27,28]. These differences must be considered when studying factors that dilate the pupil; for this purpose, researchers calculate a pupil baseline interval for each individual separately. Then, the pupil change is estimated by contrasting information from the baseline and testing intervals. In the baseline period, users fixate on a predefined screen before the stimulus is presented. Baseline duration ranges from 400 milliseconds to 10 seconds [6,29-32]. In general, the variation in the baseline duration should play no substantial role in reporting pupil dilation [33]. Unsworth et al [32] suggest that better results can be obtained by using a longer duration; hence, they use 5 seconds to estimate the baseline.

A common practice is to use a neutral image, either black, gray, or white [31,34]; a gray image is more effective to reduce screen luminescence [35]. Using a neutral image is good enough for controlled tests that use luminance-controlled images, but there are significant changes in pupil size due to luminance when participants play video games [36,37]. Studying the pupil dilation induced by mental activity when participants are exposed to environmental illumination changes is a challenge. For instance, several authors have reported that pupillary response features are directly correlated to cognitive load. Other authors, however, do not observe such correlations, and they suggest that this effect could be caused by luminance changes [38,39].

Obtaining a baseline for each trial rather than for a whole test session is a common practice [33]; this is an applicable solution for settings where the screen luminance remains stable for certain periods (eg, for a video game stage that is mainly dominated by the background). For these cases, the baseline is usually calculated from data generated by observing a scrambled image (ie, one image obtained by applying a scrambling scheme to a representative image in the period test).

Image scrambling [40] has two objectives: to transform a meaningful image into a meaningless or disordered image and to have the same mean intensity for the scrambled and original images.

The nonlinear relationship between luminance changes and pupil size is one of the main difficulties when studying cognitive load in real conditions. Wong et al [41] study four approaches (ignoring, excluding, compensating, or using pupillary light reflex features) to mitigate the luminance change in cognitive load measurements. They found that ignoring the luminance change is the worst option. This paper proposes an initial solution for studying cognitive load in real scenarios that is complementary to the approaches in the aforementioned study [41].

We hypothesize that a better baseline can be estimated from an image that maintains both the mean and local intensity. We tested grid scrambled images for obtaining the baseline. A grid scrambled image is generated by selecting a representative image within the measurement period, splitting it into a *n* × *m* grid (*n* columns and *m* rows), and finally, scrambling each region to conform the image.

The contribution of this paper is twofold: we propose a grid scramble filter to reduce the effect of screen luminescence, and we test the hypothesis that using pupillary response data improves the classification of easy (or hard) difficulty levels.

The rest of this paper is organized as follows: the Methods section describes the experimental setup, including materials, participants, metrics, and procedure; the Results section discusses the results of each experiment; and finally, the Conclusions and Further Work section concludes this paper.

## Methods

The goal of this study is to analyze the pupillary response and gamer data for different difficulty levels in a math EVG to evaluate the significant differences in perceived difficulty for participants with intermediate math skills. Selected relevant features are used to classify difficulty.

### Materials

An *eye-tracking* device, the “EyeTribe” model ET1000 with 60 Hz sampling frequency, was used in a screen (24“ extended monitor) with a resolution of 1440 × 960 pixels, and both were connected to a laptop.

The eye tracker was located 50-60 cm from the participant’s face. A calibration was done before each test/play session by using the EyeTribe software development kit (twelve points). To remove atypical values, a Hampel filter was used in the preprocessing stage.

To avoid pupil dilation caused by sunlight, the windows in the testing room were covered with blackout curtains, which have a high light-blocking effect. We used the same brightness and settings of the screen throughout. In addition, no sounds and visitors were allowed in the experimentation area.

**Figure 2:**
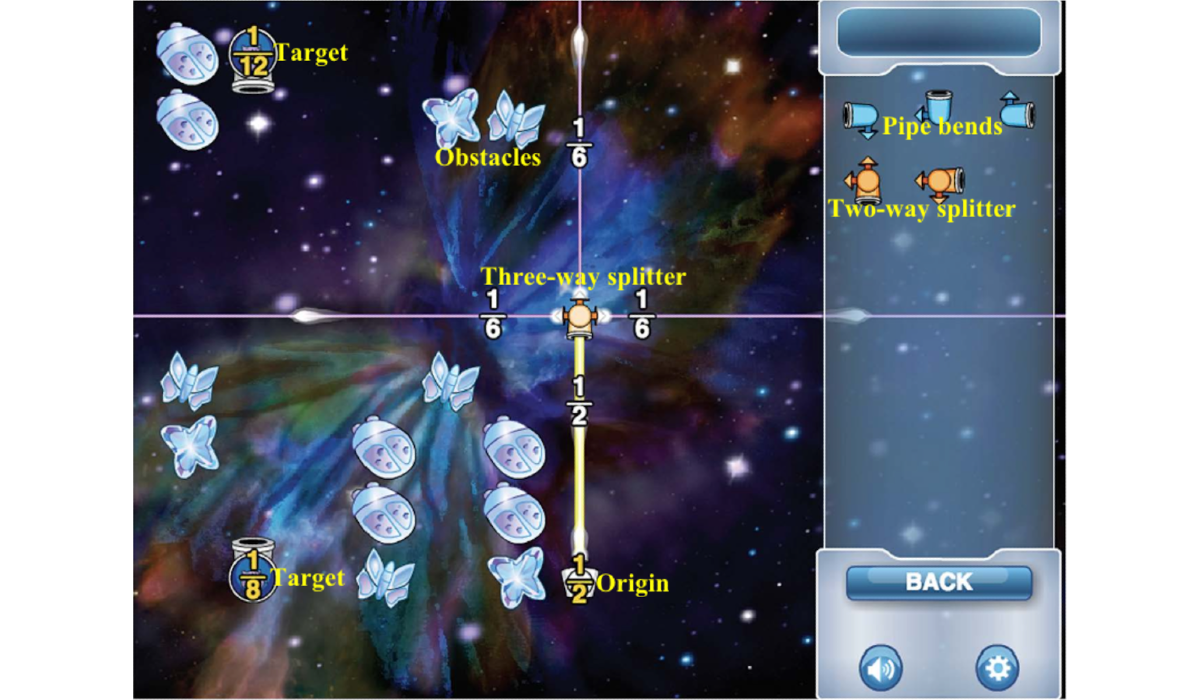
The Refraction EVG developed by the research group of the Center for Game Science [42,43]. The game mechanic is to use the pieces on the right to split lasers into fractional pieces and redirect them to the target spaceships.

The educational *Refraction video game* [42,43] was used in the experiments, as shown in Figure 2. For research, “Refraction” is of particular interest because it is open-access, it provides a natural context for students to create fractions through splitting, and the log data for the game allows the use of learning analytics methods to examine the splitting process in detail [43,44]. Moreover, the design of the game allows us to modify mathematical and game difficulty semi-independently [42].

This game focuses on teaching fractions and discovering optimal learning pathways for math learning. It let gamers bend, split, and redirect lasers to power spaceships filled with lost animals. The general integrated instruction is “Help free as many animals as you can by expanding your knowledge of fractions.” As shown in Figure 2, game elements in Refraction are *origins*, which generate laser beams; *targets*, which receive the laser beams and contain spaceships with lost animals waiting to be released; *pipe bends* that change the laser direction; *2- or 3-way splitters* that split the laser into two or three equal parts (eg, the operation of a 3-way splitter over half of a laser is ½ ÷ 3 =
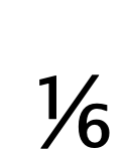
); and *obstacles* that prevent the passage of any laser beams.

Four levels of the Refraction game were selected for experiments and organized into two worlds: world A (levels *L1_a_* and *L2_a_*), and world B (levels *L3_b_* and *L4_b_*). As shown in Table 1, levels that almost have the same number of game elements were grouped into the same world (ie, *L1_a_* and *L2_a_* have about the same difficulty level).

**Table 1:** Number of game elements in the selected levels.

Element	Level			
	World A		World B	
	L1_a_	L2_a_	L3_b_	L4_b_
Origins	1	1	1	3
Targets	1	2	2	3
Two-way splitter (orange)	2	2	1	1
Three-way splitter (orange)	1	1	2	3
Pipe bends (blue)	3	3	3	3
Obstacles	10	10	13	10
Total elements	18	19	22	23

### Experiment 1

**Figure 3:**
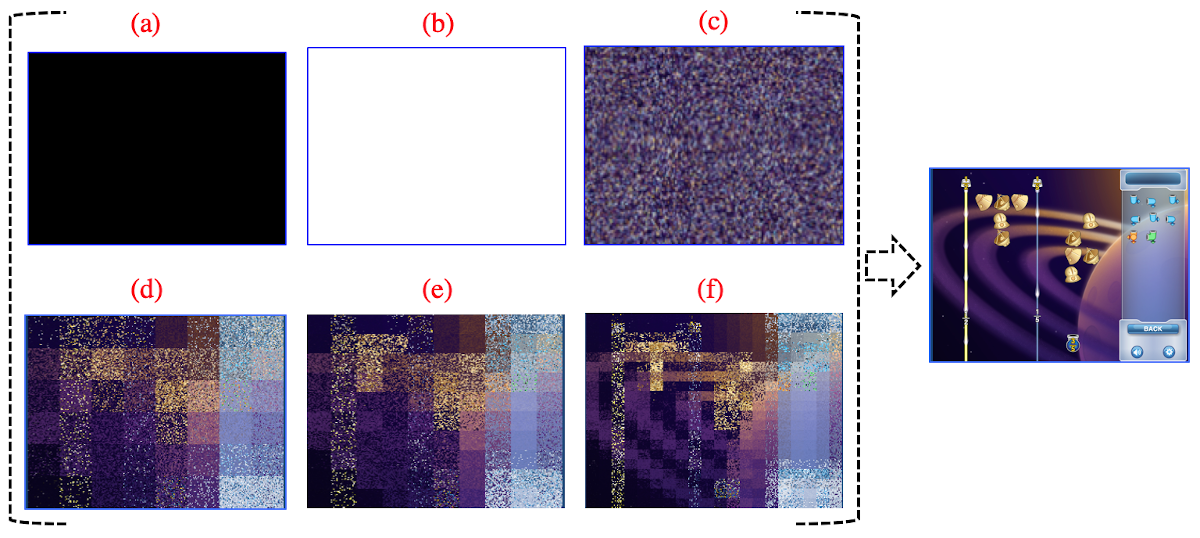
Baseline images tested. (Left) Baseline images can be uniform such as (a) black and (b) white, or can depend on the initial image like (c) scramble, (d) 8×6 grid scramble, (e) 10×10 grid scramble, and (f) 20×20 grid scramble. (Right) The in-test image.

The objective of this experiment was to select the best baseline image (ie, a baseline image without semantic information that results in a smaller pupil-size change after the transition from the baseline image to the in-test image). Instances of tested baseline images are shown in Figure 3; they included the widely used white, black, and scramble backgrounds, but also grid scramble images of different sizes: 8×6, 10×10, and 20×20.

#### Participants

All participants were asked about their general health and were excluded if they wore contact lenses or glasses with more than one power, had eye surgery or abnormalities (eg, lazy eye, strabismus, nystagmus), or used medication or drugs. All participants were Hispanic and brown-eyed. Participants were not asked for personal information to preserve anonymity. A total of 14 volunteers (4 female, 10 male) between 16 and 37 years old (mean 21.81, SD 7.2) participated in this experiment.

#### Procedure

**Figure 4:**
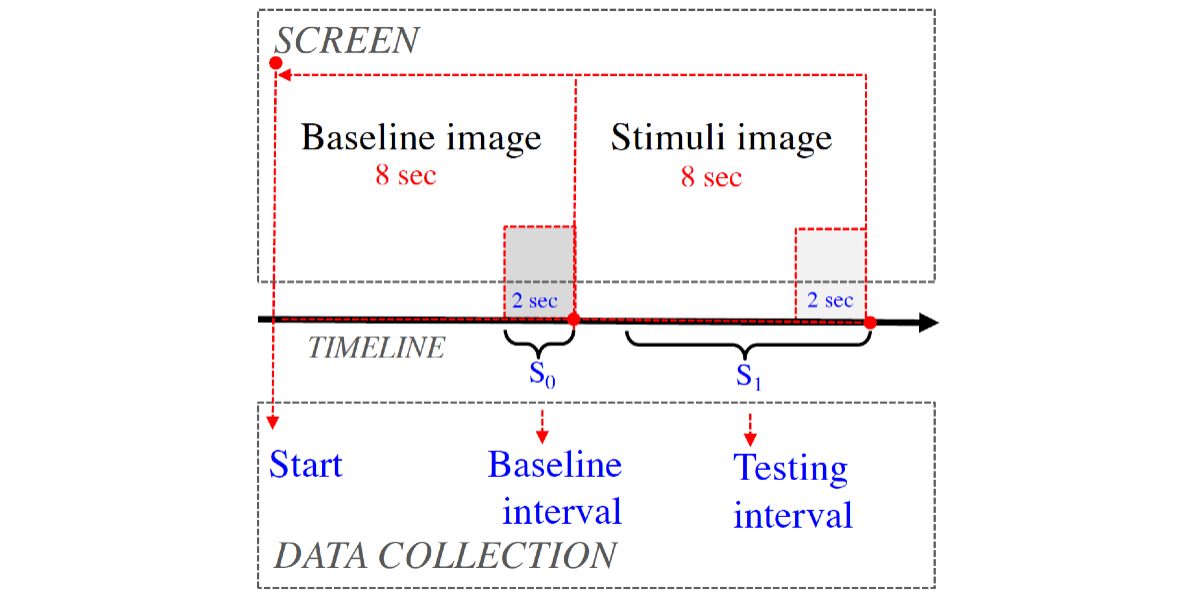
The procedure used to generate pupillary response data for evaluating baselines images. First, the baseline image was shown on the screen for 8 seconds, and then the in-test image was shown.

As illustrated in Figure 4, participants observed a randomly selected baseline image (an image from Figure 3) for 8 seconds (pupillary response data collected in the last 2 seconds are used as the baseline interval), and then they observed the in-test image for 8 seconds (pupillary data from the last 2 seconds are used as the testing interval).

The MPDC is used to select the best baseline image (the MPDC definition is shown in Table 2). This procedure was repeated until all the baseline images were shown to participants.

**Table 2:** Pupillary and gamer features studied in this experiment.

Feature	Description
TE	*Total errors* (TE) is the number of events performed in the wrong way (eg, the laser beam value does not match with the input value) on a level.
TC	*Time to complete a stage* (TC) is the time required to complete a given level.
CP	*Number of changes of position* (CP). A *change of position* is defined as the movement of a game element once it has been introduced in the gameplay—the area where the video game elements are dragged and dropped.
A	*Attempts* (A) is the number of attempts used by the gamer to complete a given level.
MPDC	The *mean pupil diameter change* is obtained by averaging the relevant data points in the measurement interval (time of the stage) and subtracting the mean diameter obtained in the baseline period [24-26].
PD	*Peak dilation* (PD) is defined as the maximal dilation obtained in the measurement interval time of the level [13]. First, mean baseline is established, then the single maximum value from the set of data points in the measurement interval time of level is selected.
LP	*Latency to peak* (LP) reflects the amount of time elapsed between the beginning of the measurement interval and emergence of peak dilation [13].
APCPS	*Percentage change in pupil size* (PCPS) is calculated as the difference between the measured pupil size and a baseline pupil size divided by the baseline pupil size [22,31,45]. The *average PCPS* (APCPS) is the average of PCPS in the measurement interval time of the selected level.

#### Statistical Analysis

After Mauchly's test of sphericity, repeated-measures analysis of variance was performed on the normally distributed variables among MPDC values to explore the difference between the black, white, scramble, scramble 8×6, scramble 10×10, and scramble 20×20 baseline images. The Bonferroni test was used to make post hoc pairwise comparisons.

### Experiment 2

The objective of this experiment was twofold: to evaluate which features are more related to the difficulty level, and to test the classification accuracy obtained by using different subsets of features. Studied features (both pupillary and gamer) of the video game levels (L1_a_, L2_a_, L3_b_, and L4_b_) are defined in Table 2.

#### Participants

A total of 20 volunteers (9 female, 11 male) between 23 and 31 years old (mean 27.16, SD 2.6) participated in experiment 2. As in the first experiment, we did not include volunteers with some characteristics that would make pupil-size estimation difficult. None of the subjects who participated in experiment 2 also participated in experiment 1.

#### Procedure

**Figure 5:**
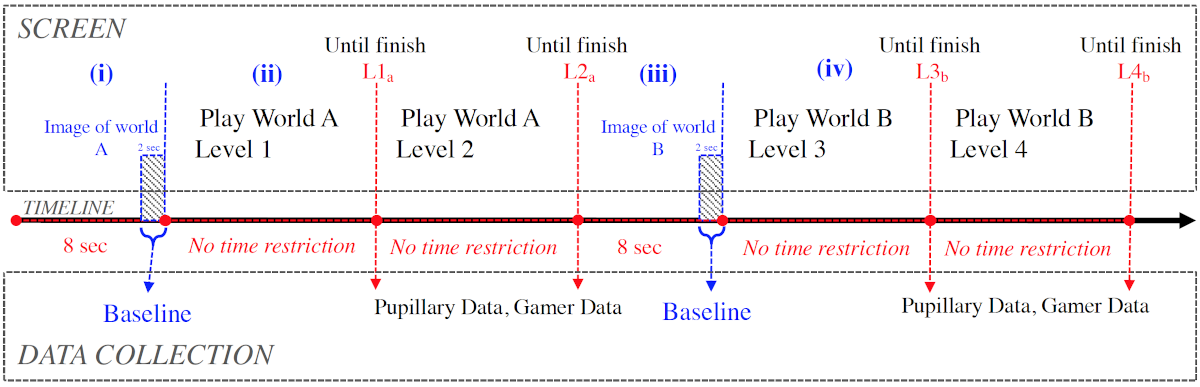
The procedure used to evaluate features against difficulty levels in World A (easy), World B (hard).

As shown in Figure 5, the procedure consists of four phases: (1) participants observed the baseline image of world A for 8 seconds; (2) participants played the world A levels (L1_a_ and L2_a_) without time restrictions; (3) participants observed the baseline image of world B for 8 seconds; and finally, (4) they played the world B levels (L3_b_ and L4_b_) without time restrictions. The pupil baseline was estimated from the data of the last 2 seconds before playing a new world. Pupil size and gamer behavior data were collected along with each play session.

After obtaining features, all information was integrated into a data set *τ = {(X_i_, Y_i_), i = 1,...,n}*, where *X_i_* corresponds to the uniform-length vector containing features *X_i_ = (TE_i_, TC_i_, CP_i_, A_i_, MPDC_i_, PD_i_, LP_i_, APCPS_i_)* and *Y_i_* corresponds to the label associated to each level difficulty of the world A and world B. Each register of this data set is generated from a player and a single level. The following sets were defined: *G = {TE, TC, CP, A},* which includes all game behavior data features, and *S = {MPDC, PD, LP, APCPS},* which includes all pupillary features. Let *G’*⊆*G* and *S’*⊆*S* be the sets of features with a significant difference between worlds A and B.

From the 20 participants, 3 (15%) were randomly selected, and their registers in *τ* were used to train a random forest classifier [46] using different sets of features. Random forest classifier was selected because it is an ensemble meta-algorithm that improves accuracy and avoids overfitting by training on different random samples of the data. Registers in *τ* associated with the rest of the participants were then used as the testing set.

#### Statistical Analysis

Features were tested for normality; in this case, the Shapiro-Wilk test was used (because of the low size of the sample), Results show that the variables are not normally distributed. Then, the Wilcoxon signed rank test was used to detect significant differences in variables. Differences between values were considered significant when *P*<.05.

## Results

### Experiment 1

Mauchly's test of sphericity indicated that the assumption of sphericity had been violated (*χ*^2^_14_=0.05; *P*<.01); therefore, a Greenhouse-Geisser correction was used (*ε*=0.47). The results show that there was a significant difference between MPDC estimated from different baseline images (*F*_5,78_=30.965; *P*<.001).

Table 3 shows the descriptive statistics for MPDC calculated for each baseline image. As expected, the 20×20 scrambled filter has the lowest average MPDC (0.32 pixels) as it more closely resembles the original image. Post hoc analyses using the Bonferroni post hoc criterion for significance indicated that there were no MPDC differences for different grid sizes, but there were significant MPDC differences between the group of images generated by the grid scrambled filter, and the group of conventional images used to estimate the baseline (white, black, and scrambled). We choose the 8×6 grid scramble operation for generating baseline images in experiment 2 because there are no differences in MPDC between grid scramble images, and it better obscures the meaning of the in-test image.

**Table 3:** Results for the baseline image test (experiment 1). Different superindices indicate significant intergroup differences.

Baseline image	MPDC^a^ (pixels), mean (SD)
White^1^	3.356 (2.122)
Black^2^	−1.754 (1.452)
Scramble^3^	1.620 (0.746)
Grid scramble 8×6^4^	0.471 (0.891)
Grid scramble 10×10^4^	0.455 (1.392)
Grid scramble 20×20^4^	0.320 (0.856)

^a^MPDC: mean pupil diameter change.

### Experiment 2

We did not find any feature with significant differences in measurements between levels of the same world, neither in the levels of world A (L1_a_, L2_a_) nor in the levels of world B (L3_b_, L4_b_). However, significant differences between worlds were found for the following features: TE between world A (median 0.00) and world B (median 2.50) (*z*=−2.9; *P*=.004); TC between world A (median 43,486) and world B (median 83,970) (*z*=−3.198; *P*=.001); MPDC between world A (median 2.25) and world B (median 2.90) (*z*=−2.159; *P*=.03); and PD between world A (median 5.1) and world B (median 18) (*z*=−3.587; *P*<.001). Table 4 summarizes the statistics for pupillary and gamer features and the Wilcoxon signed rank results.

On the other hand, Table 5 summarizes the accuracy of the random tree classifier. As can be seen, the PD feature alone gives an accuracy of 62.5%. The best accuracy was obtained by using the *G’* ∪ *P’* features, with an accuracy of 87.5%.

**Table 4:** Median values for pupillary and gamer measurements, and the Wilcoxon signed rank results.

Feature	World A, median	World B, median	*z*	*P* value
TE	0.00	2.5	−2.900	.004
TC	43,486	83,970	−3.198	.001
CP	0.00	1.00	−0.382	.70
A	0.50	1.00	−0.282	.78
MPDC	2.25	2.90	−2.159	.03
PD	5.10	18.00	−3.587	<.001
LP	40.50	51.50	−0.973	.33
APCPS	0.135	0.136	−0.926	.36

**Table 5:** Results for a random forest classifier using different sets of features.

Set	Features	Accuracy (%)
*G*	TE, TC, CP, A	75.0
*G’*	TE, TC	75.0
*P*	MPDC, PD, LP, APCPS	50.0
*P’*	PD	62.5
*G’* ∪ *P’*	TE, TC, PD	87.5

## Discussion

### Experiment 1

Pupil-size changes at the beginning of the EVG (when going from the baseline image to the in-test image) can cause the participant's pupil to expand. A change caused by the screen luminescence would hide the change caused by the cognitive load produced by the reasoning task. This change was analyzed using the MPDC in experiment 1; it was found that baseline images with uniform colors (white and black) result in larger changes in pupil size (Table 3). The sign values of the MPDC are aligned with the optics of the human eye, as it is posited that pupil size increases when the intensity of environmental light decreases (in the case of black or white images); these changes occur even if baseline images resembles the general illumination conditions of the testing scenario such as the scrambled operation.

One could expect that a grayscale image, with the same average intensities as the in-test images, gives a good baseline estimator. Results of experiment 1 show that the conventional scrambled image (which has about the same intensities) just gives a rough estimation of the baseline. Alternatively, the proposed grid scrambled operation better estimates the baseline in comparison to the conventional scramble image. A possible explanation is that retinal ganglion cells (the output neurons of the retina) adapt to both image contrast (the range of image intensities) and to spatial correlations within the scene, even at constant mean intensity [47]. Hence, predicting the pupil size of an individual in different image scenes is challenging. John et al [48] propose a calibration protocol where the participant sees uniform slides of varying grayscale intensities in the range 0-255. We state that a better model could be found by using local and global information from the images.

### Experiment 2

Many studies have shown that splitting objects is a promising way to teach fractions [43,49]. In any context, splitting items into halves is much more common than dividing into thirds; this could explain why the students prefer halving and struggle with creating thirds [43]. The Refraction game uses the process of splitting to teach fractions. As shown in Table 1, levels of world A (easy) have fewer 3-way splitters than levels of world B (hard). This means that participants must solve more operations that involve thirds in world B. The difficulty of the Refraction game not only depends on the mathematical operations but on the spatial difficulty. The spatial difficulty is directly correlated to the number of sources and targets; the number of source/target elements is smaller in the world A than in the world B. Results also evidence this change of difficulty, as we observed statistical differences in features G’—including TE and TC.

A random tree classifier that only uses the best game features, G’, only gives an accuracy of 75.0%. This accuracy was improved to 87.5% by using the *peak dilation.* The maximal dilation obtained in the measurement interval is a natural feature of many factors that dilate the pupil, including the cognitive load.

Pupillary features can be classified into subtractive (those that eliminate individual differences by subtracting the baseline value from the measurement interval, such as *MPDC, PD,* and *LP*) and divisive (those that calculate a ratio of a measurement value to baseline, such as *APCPS*). Subtractive features can be categorized into *size-related*, such as *MPDC* and *PD*, or *time-related*, such as *LP*. Results show that the subtractive size-related features, *MPDC* and *PD,* better describe the difficulty level.

Hunicke [50] states that difficulty adjustments must be implemented in a way such that users do not perceive difficulty changes. However, gamer data are recorded after human perception of difficulty; that is, a control that uses gamer data collected after the player finished each level could not completely fulfill the requirement of being imperceptible.

The proposed approach improves the accuracy of classification of the perceived difficulty to 87.5%, in contrast to 62% with only pupillometry. These results are aligned to other studies that suggest the relationship between pupil change and the level of a game; for instance, by using the Akaike Information Criterion, Strauch et al [51] propose that the pupil change is a quadratic function of the levels of Pong.

Video game difficulty adjustment is game data–dependent (ie, different games require different features). We argue that a generic framework for dynamic difficulty adjustment could be designed by fusing generic game features (such as score, elapsed time, etc) with the information provided by pupillometry. In this way, we can take advantage of ocular data as a general, noninvasive, near real-time option to sense the user perception of difficulty.

In a traditional pupillometry experiment, the researcher maintains tight control over luminance while manipulating a specific cognitive variable. Reilly et al [52] conducted the reverse approach (ie, holding cognitive task demands constant while manipulating luminance). We believe that the reverse approach must be used to obtain a model of the participants’ pupil size in the initial calibration stage by using the grid scrambled images, and then a subtractive approach should be used during the gameplay stage.

### Conclusions and Further Work

This paper proposes a grid scramble filter to obtain a baseline image that reduces the effect of the screen light reflex on a participant's pupil size. This filter simulates both the local and the mean luminance of a given image. To hide the meaning of an image, the 8×6 grid scramble filter can be used for tests that reasonably keep the same background in each interval. We consider that a more general baseline can be obtained by modeling luminescence factors that affect pupil size. Such a model could be used to estimate cognitive factors that affect the pupils in any setting (eg, a commercial video game).

Gamer data are a valuable resource for estimating the difficulty of EVGs, but adding cognitive load data measured by pupillary response data improves the accuracy of classifying the difficulty of game levels.

Using the human perception features from ocular data such as blinks, eye-fixations, and eye-saccade to measure the cognitive load may improve the classification accuracy of difficulty levels and gather imperceptible changes that gamer data can omit [53,54].

A key issue with approaches that estimate a baseline, like the proposed one, is that indoor light conditions and monitor brightness must be the same during the game time. Playing a game in specific conditions is restrictive; to address this, we are working on a model that relates luminescence to different screen configurations (instead of a baseline) This approach can be used in virtual reality headsets. The proposed approach can be included in a more elaborated calibration stage that tests different models of pupil change due to luminance, as in a previous study by Lara-Alvarez and Gonzalez-Herrera [55].

## Abbreviations

A: attempts
APCPS: average percentage change in pupil size
CP: number of changes of position
EVG: educational video game
LP: latency to peak
MPDC: mean pupil diameter change
PD: peak dilation
TC: time to complete a stage
TE: total errors
TEPR: task-evoked pupillary response
